# Cytokeratin 7 and 20 staining for the diagnosis of lung and colorectal adenocarcinoma

**DOI:** 10.1038/sj.bjc.6600326

**Published:** 2002-06-17

**Authors:** S Kummar, M Fogarasi, A Canova, A Mota, T Ciesielski

**Affiliations:** Department of Medicine, Yale Cancer Center, Yale University School of Medicine and VA Connecticut Cancer Center, VA Connecticut Healthcare System, 950 Campbell Avenue, 111D, West Haven, Connecticut, CT 06516, USA; Department of Pathology, VA Connecticut Healthcare System, West Haven, Connecticut, USA

**Keywords:** cytokeratin staining, lung adenocarcinoma, colorectal cancer, adenocarcinoma of unknown primary

## Abstract

The origin of metastatic adenocarcinoma lesions can sometimes be difficult to diagnose. The objectives of our study were to establish the cytokeratin staining pattern of primary and metastatic lung and colorectal adenocarcinomas, and to determine if this helps to identify the site of origin of metastatic lesions. We reviewed a total of 102 tissue samples from patients in our tumour registry, with either primary or metastatic lung or colorectal adenocarcinoma. Tissue sections were stained for cytokeratin 7 and 20 and read as positive or negative for staining. Clinical and radiologic information was reviewed from computerised charts. The cytokeratin 7+/cytokeratin 20− pattern characterised 96% (29 out of 30) of primary and 95% (21 out of 22) of metastatic lung adenocarcinomas. All the primary (26), and 88% (21 out of 24) of metastatic colorectal adenocarcinomas stained cytokeratin 7−/cytokeratin 20+. Samples from a variety of metastatic sites were evaluated for cytokeratin 7 and 20 staining. Out of the 102 samples, in 95% (97 out of 102) of the cases, the cytokeratin 7 and cytokeratin 20 staining pattern characterised and differentiated between lung and colorectal adenocarcinoma. Primary and metastatic lung adenocarcinomas show a cytokeratin 7+/cytokeratin 20− staining pattern, while colorectal adenocarcinomas stain cytokeratin 7−/cytokeratin 20+. Cytokeratin staining is helpful in the diagnostic differentiation of metastatic lesions from these two common primaries, and assists in determining the site of origin of metastatic lesions.

*British Journal of Cancer* (2002) **86**, 1884–1887. doi:10.1038/sj.bjc.6600326 www.bjcancer.com

© 2002 Cancer Research UK

## 

In patients who present with metastatic disease, determining the primary site of origin may have a major impact on prognosis and therapy. However, in spite of clinical, radiographic, and routine histologic studies, the primary site remains uncertain in approximately 3–15% of cases (Abbruzzese *et al*, 1995; Hammar, 1998). A significant proportion of these cases are adenocarcinomas, and they present a challenge for the pathologist and the oncologist alike.

Cytokeratins are intermediate filament proteins present in epithelial cells. They are expressed in a tissue-specific manner in normal organs and the tumours that arise from them. Monoclonal antibodies have been established against several of the cytokeratin polypeptides and are used to evaluate the pattern of cytokeratin expression in cells of epithelial origin. Chu *et al* (2000) studied the expression of two of the low molecular weight cytokeratin subtypes, CK7 and CK20, in 435 cases of epithelial neoplasms. They observed CK7 expression in the majority of lung, breast, endometrium, ovary, cervix, salivary gland, thyroid cancers, cholangiocarcinoma, and adenocarcinoma of the pancreas. In contrast, CK20 staining was seen in all colorectal adenocarcinomas and in the majority of gastric, pancreatic, and Merkel cell tumours. Prostate, kidney, and adrenocortical carcinomas as well as sarcomas, carcinoids, hepatocellular carcinomas and thymomas were negative for both CK7 and CK20 staining, while a large proportion of pancreatic and cholangiocarcinomas as well as some bladder and gastric tumours expressed both CK7 and CK20.

Based on the differential pattern of cytokeratin staining reported in the literature, we decided to evaluate the cytokeratin expression in tissue samples of lung and colorectal adenocarcinomas, obtained from our patient population. The objectives of our study were to: (i) establish the cytokeratin staining pattern of primary lung and colorectal adenocarcinomas; (ii) correlate the expression of the primary with that of the metastatic lesion; and (iii) determine if evaluating the cytokeratin 7 and 20 staining helps to identify the primary site of origin of metastatic lesions.

## MATERIALS AND METHODS

One hundred and two samples from patients with primary or metastatic lung or colorectal adenocarcinoma were identified from the Veteran's Administration (VA) Connecticut Cancer Center Tumor Registry (1995–2001) and evaluated for cytokeratin staining. All patients were male, representing a relatively homogenous population with regards to age and risk factors for lung cancer, namely smoking. All tumour samples were adenocarcinomas, with most being moderately to poorly differentiated. The sites of metastatic disease biopsied in patients with lung cancer included the pleura, pleural effusion, soft tissue, bone, small bowel, adrenal gland, bone marrow and liver. Metastatic colorectal cancer deposits that were sampled included deposits in the lung, liver, lymph nodes, chin and small bowel. Clinical information was reviewed from computerised charts.

Tissue blocks and cytology specimens were obtained and slides cut at 5 microns. Sections were then mounted on positive charged slides Shandon catalogue number 6776214, Shandon Inc., Pittsburgh, PA, USA and placed in a laboratory incubator at 60°C, for 40 min. Slides were cooled and re-hydrated with three changes of Histosolve (Shandon catalogue number 6764508) for 5 min each, two changes of absolute ethanol for 5 min each, one change of 95% ethanol for 5 min, and then transferred to tap water. Slides were then ‘quenched’ in a mixture of methanol and 3% hydrogen peroxide (97 ml methanol with 3 ml of 3% hydrogen peroxide added) for 30 min.

Slides were then placed on an automated immunohistochemistry stainer (Ventana ES, Ventana Medical Systems, Tucson, AZ, USA) with the following reagents: Mouse anti-human cytokeratin 7 monoclonal antibody (Dako catalogue number M 7018, Dako Corporation, Carpinteria, CA, USA), 1 : 200 dilution using Ventana antibody diluent (catalogue number 251-018) for 34 min; mouse anti-human cytokeratin 20 monoclonal antibody (Dako catalogue number M 7019), 1 : 10 dilution using Ventana antibody diluent (catalogue number 251-018) for 34 min. Subsequently, the slides were processed with a DAB detection kit (Ventana catalogue number 250-001) and counterstained using haematoxylin (Ventana catalogue number 250-2021) for 8 min, followed by immersion in bluing reagent (Ventana catalogue number 250-2037) for 4 min.

Slides were removed from the stainer and washed with tap water for 10 min. They were first placed in 95% ethanol and then in absolute ethanol for 5 min each, followed by four changes of Histosolve for 5 min each. They were subsequently coverslipped using a synthetic mounting media (Permount, Fisher catalogue number SP-15-100, Fisher Healthcare, Houston, TX, USA).

Positive and negative controls were used as a basis for scoring the sample slides. Immunostaining was evaluated semiquantitatively by estimating the number of stained cells. Samples with over 5% of tumour cells showing CK staining were considered positive (Wauters *et al*, 1995). At least two reviewers (T Ciesielski and S Kummar) reviewed all slides.

## RESULTS

A total of 102 samples of adenocarcinoma were identified from our tumour registry and evaluated for cytokeratin expression using cytokeratin 7 and 20 immunostains. Cancer cells were identified morphologically and assessed for staining. Routine background staining for CK7, but not for CK20, was observed for normal mesothelial and alveolar cells in all lung biopsy slides, as has been previously described in the literature. Normal colonic mucosa, as well as benign polyps in the colon, were consistently strongly positive for CK20 staining. No background staining of normal colorectal mucosa with CK7 was observed.

### Primary lung and colorectal adenocarcinoma

**Figure 1:**
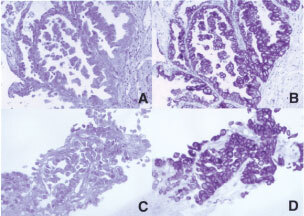
CK20 (**A**) and CK7 (**B**) immunostaining of primary lung adenocarcinoma. CK20 (**C**) and CK7 (**D**) immunostain of metastatic lung adenocarcinoma to the liver. Most of the tumour cells in both the primary and the metastatic lesion show strong cytoplasmic staining with CK7 and negative staining with CK20 (original magnification 200×).

**Figure 2:**
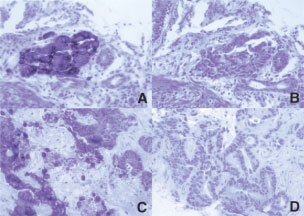
CK20 (**A**) and CK7 (**B**) immunostaining of primary colon adenocarcinoma. CK20 (**C**) and CK7 (**D**) immunostain of metastatic colon cancer to liver. There is focal strong cytoplasmic staining for CK20 in both the primary and the metastatic lesion and negative staining with CK7 (original magnification 200×).

In the initial phase of the study, the pattern of cytokeratin staining for primary lung and colorectal adenocarcinoma was evaluated. A total of 30 primary lung and 26 primary colorectal lesions were studied. All primary lung lesions were CK7+, and 29 of these were CK20− (Figure 1

). In one case, focal CK20 staining of some of the adenocarcinoma cells was observed. This patient was found to have two tubular adenomas, with no evidence of a malignant lesion, on a surveillance colonoscopy, 8 months prior to his lung surgery. All the primary colon lesions (26) were CK7−/CK20+ (Figure 2

).

Overall, the CK7+/CK20− staining pattern was observed for 96% of primary lung adenocarcinoma lesions, while 100% of the colorectal cancers showed the CK7−/CK20+ staining pattern (Tables 1

**Table 1:**
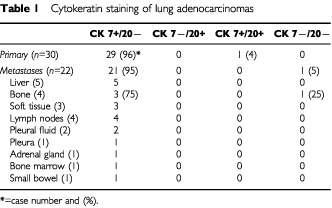
Cytokeratin staining of lung adenocarcinomas

and 2

**Table 2:**
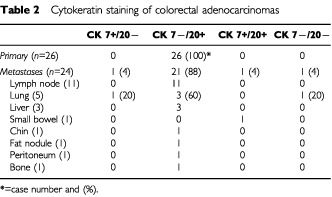
Cytokeratin staining of colorectal adenocarcinomas

). We did not find consistent differences in the staining intensity or per cent positive cells based on grade of the tumour.

### Patients with metastatic disease and/or recurrent disease

#### Patients with advanced metastatic adenocarcinoma and radiologic evidence of a lung mass

The metastatic site, and not the radiologically apparent lung mass, was biopsied and a total of 22 samples were studied. These included sites involving the pleura, pleural effusion, soft tissue, bone, small bowel, adrenal gland, bone marrow and liver. Ninety-five per cent of the samples stained CK7+/CK20−, consistent with a lung primary. No consistent difference in staining pattern was observed for the different metastatic sites. One case, involving the rib, stained negative for both CK7 and CK20. The corresponding lung primary stained positive for CK7, and negative for CK20. The other three samples of metastatic lung cancer involving the bone, stained consistently CK7+/CK20−. In five cases, the metastatic site was biopsied prior to the radiologic imaging of the chest, thus, helping to guide further diagnostic evaluation (Table 1).

#### Patients with a known history of colorectal cancer, who subsequently presented with metastatic adenocarcinoma

A total of 24 sites of recurrence/metastasis were biopsied and evaluated for patterns of cytokeratin staining. These metastatic sites included deposits in the lung, liver, lymph nodes, chin and small bowel. Twenty-one (88%) samples revealed the CK7−/CK20+ staining pattern (Figure 2). In six cases, cytokeratin staining of the original colon tumour was also obtained, and stained CK7−/20+. Of the three cases that did not show the CK7−/CK20+, in one case the lung lesion was CK7+/CK20−, consistent with a second primary, arising in the lung. In the second case, the lung biopsy stained negative for both CK7 and CK20. In this patient, the primary colon tumour was CK7−/CK20+, but the CK20 staining was focal. In the third case, the patient had a small bowel mass, felt to be a metastasis from the known colorectal primary. Biopsy of this mass stained positive for both CK7 and CK20.

Overall 102 samples were tested, and in 95% of the cases, the CK7/CK20 staining pattern characterised and differentiated between lung and colorectal adenocarcinoma.

## DISCUSSION

There may be both prognostic and therapeutic information in determining the site of origin of metastatic lesions. Our VA patient population represents a relatively homogenous group of patients with regard to age and sex, as well as their risk factors for both lung and colorectal cancers. As a result, in patients who present with metastatic adenocarcinoma, it is important to determine whether the primary arose in the lung *vs* the colon.

Cytokeratins, a family of over 20 polypeptides, are markers of epitheloid differentiation. The various cytokeratin subtypes are expressed in a tissue-specific manner on cells of epithelial origin. Adenocarcinomas of the lung have been reported to be CK7+, while colon cancers are positive for CK20 staining (Wang *et al*, 1995; Rubin *et al*, 2001). Carcinoembryonic antigen (CEA) expression can also be used to evaluate colon adenocarcinoma lesions (Lagendijk *et al*, 1998, 1999). However, primary lung cancer also commonly stains positive for CEA, making the differentiation between colon and lung primaries difficult based on CEA expression alone (Raab *et al*, 1993). In the study presented herein, we decided to evaluate the cytokeratin staining pattern of adenocarcinoma samples from patients with primary and metastatic lung and colorectal cancer. We wanted to determine if both the primary and metastatic lesions stain consistently for CK7 and CK20, and whether this aids in the diagnosis of the primary site of origin of the metastatic lesions.

First, we established the CK7 and CK20 staining pattern of primary lung and colorectal adenocarcinomas. Ninety-six per cent of the primary lung lesions were CK7+/CK20−, while 100% of colorectal primaries stained CK7−/CK20+. No differences were observed in the staining pattern of right- *vs* left-sided colon lesions or rectal primaries. There also was no significant inter-observer difference, although the study did not set out to establish the reproducibility of CK staining.

Based on the above results, we decided to evaluate the staining pattern of metastatic adenocarcinoma lesions at a variety of sites from patients with either evidence of a dominant lung mass by radiologic studies or known history of colorectal cancer. The CK7+/CK20− pattern was observed for 95% of metastatic lesions biopsied in patients with primary lung cancer. One sample obtained from the rib, in a patient with a lung mass, stained negative for both cytokeratin 7 and 20. The primary lung tumour from this patient showed strong staining with CK7, and was negative for CK20. The lack of staining in the rib lesion could be due to sampling error, as the amount of tumour tissue in the biopsy specimen was small. We had three more cases of lung cancer metastatic to bone, two involving the ribs and one in the femur. All three showed strong and consistent staining with CK7. In patients with a prior history of colorectal cancer, 88% of the samples showed the characteristic CK7−/20+ staining pattern, consistent with metastasis from the known cancer. Thus, in our study we found a consistent pattern for both the primary and metastatic lesions, with no evidence of loss of cytokeratin staining in synchronous or metachronous metastatic lesions. Loy and Calaluce (1994) evaluated the cytokeratin 7 and 20 staining patterns of 50 metastatic lung or colon adenocarcinoma samples. They reported that 83% of metastatic lung lesions showed CK7+/CK20− staining, while 69% of metastatic colorectal lesions retained the typical CK7−/CK20+ staining pattern. Unlike in our series however, in their group, none of the metastatic lung lesions and only two of the colorectal metastatic deposits were in the liver.

Liver is a common site of metastasis, making the diagnosis of primary site of origin difficult. In a large autopsy series of adenocarcinomas metastatic to the liver, reported by Tot (1999), staining for both CK7 and CK20 was evaluated. The CK7−/CK20+ pattern was observed for 17 of the 21 cases of colorectal adenocarcinoma. However, no cases of primary lung adenocarcinoma were included in this series. We identified and evaluated eight samples from liver biopsies representing metastatic adenocarcinoma in our study population. In five of the cases, the cytokeratin staining pattern was CK7+/CK20−. Subsequent diagnostic imaging studies revealed the presence of a dominant lung mass, consistent with primary lung cancer. In the other three cases, the patients had history of colon cancer and subsequently presented with liver masses. Liver biopsy samples in all these cases stained CK7−/CK20+, consistent with metastatic colorectal cancer.

Patients with history of colorectal cancer, who present with an isolated adenocarcinoma lesion without evidence of other sites of metastasis, raise concerns regarding whether this represents a second primary *vs* metastasis from the known colorectal cancer. This distinction carries obvious diagnostic and therapeutic implications. In this study, we identified patients with a history of colorectal cancer, who subsequently developed a solitary adenocarcinoma lesion, without evidence of widespread metastasis by computed tomography (CT). We reviewed four cases, three had lung lesions, while one presented with a lesion in the soft tissue of the chin. Two of the lung lesions and the lesion in the chin showed a staining pattern consistent with primary lung adenocarcinoma, a second primary. Thus, cytokeratin staining of the metastatic lesion provided important information regarding site of origin. Tan *et al* (1998) reported a series of 10 cases with lung adenocarcinomas, and nine cases of colon adenocarcinoma metastatic to the lung. Biopsies from all 10 patients with lung primaries stained CK7+/CK20−, while all metastatic lesions from the colon were CK7−/CK20+ (Tan *et al*, 1998).

A large variety of sites were evaluated and showed adequate staining. One of the main advantages of using cytokeratin staining is the ability to obtain reliable staining, even when a small amount of tissue is available, as in the case of cytology specimens. Harlamert *et al* (1998) reported a series of 21 cytology samples from primary lung adenocarcinomas. All samples stained CK7+, with an average of >80% of cells showing strong cytoplasmic staining. In the present series, both solid (lung, liver and rectum) and fluid cytology (pleural fluid) samples were tested and showed strong staining for either CK7 or CK20. Moreover, repeated sampling and testing in a patient with malignant pleural effusion consistently showed the CK7+/CK20- staining pattern.

One of the drawbacks of cytokeratin staining is that tumours may stain for both CK7 and CK20. In our study population, while most tumours stained positive for either CK7 or CK20, there was one case (4%) of primary lung cancer and one case (4%) of metastatic deposit in the small bowel from a known colorectal cancer, that expressed both CK7 and CK20. The ratio of CK7/CK20 staining for lung biopsy was 10–20/1, while for small bowel it was 1/10–20. Staining for both CK7 and CK20 has been reported in the literature for some tumours (Loy and Calaluce, 1994). One of the adenocarcinomas that has been reported to be positive for both CK7 and CK20 expression is mucinous bronchioloalveolar adenocarcinoma of the lung. Goldstein and Thomas (2001) evaluated the expression of CK7 and CK20 in 14 cases of mucinous bronchioloalveolar adenocarcinomas (BAC) and 11 cases of mucinous colon adenocarcinomas. Eleven of the 14 (79%) mucinous BACs stained positive for CK20, while all 14 (100%) stained positive for CK7. All cases of mucinous colon adenocarcinoma showed staining for CK20, while only one out of 11 (9%) was positive for CK7 (ref). Therefore, both CK7 and CK20 staining of lung lesions should be obtained and can help to differentiate between a primary lung *vs* metastatic colon adenocarcinoma. In difficult cases, staining for thyroid transcription factor and villin can be helpful in differentiating mucinous BACs from mucinous colon adenocarcinomas (Goldstein and Thomas, 2001).

The causes for false negative cytokeratin staining include tumours that express low levels of CK7 or CK20, sampling error in tumours that are focally positive, and poorly differentiated or necrotic tissue that may stain poorly for cytokeratin. In our series, one case of lung lesion in a patient with metastatic colon carcinoma did not show staining with either CK7 or CK20. We reviewed the staining pattern of the primary colon cancer in this patient. It showed focal staining for CK20 (5–25% of the cells), and was negative for CK7. Thus, in this case, sampling error could have accounted for the lack of CK20 staining observed for the lung lesion.

In conclusion, we observed characteristic cytokeratin staining patterns for both primary and metastatic lung and colorectal adenocarcinoma in a series of 102 tumour samples. The CK7/CK20 pattern was able to identify lung *vs* colorectal adenocarcinoma in 95% of the cases analysed. In the correct clinical setting, cytokeratin staining is a cost effective screening tool to help in the diagnostic differentiation of metastatic lesions from these two common primary sites.
